# The proximal centriole-like structure maintains nucleus–centriole architecture in sperm

**DOI:** 10.1242/jcs.262311

**Published:** 2024-09-06

**Authors:** Danielle B. Buglak, Kathleen H. M. Holmes, Brian J. Galletta, Nasser M. Rusan

**Affiliations:** Cell and Developmental Biology Center, National Heart, Lung, and Blood Institute, National Institutes of Health, Bethesda, MD 20892, USA

**Keywords:** Sperm, HTCA, PCL, Centriole, Spag4, Yuri

## Abstract

Proper connection between the sperm head and tail is critical for sperm motility and fertilization. Head–tail linkage is mediated by the head-tail coupling apparatus (HTCA), which secures the axoneme (tail) to the nucleus (head). However, the molecular architecture of the HTCA is poorly understood. Here, we use *Drosophila* to investigate formation and remodeling of the HTCA throughout spermiogenesis by visualizing key components of this complex. Using structured illumination microscopy, we demonstrate that key HTCA proteins Spag4 and Yuri form a ‘centriole cap’ that surrounds the centriole (or basal body) as it invaginates into the surface of the nucleus. As development progresses, the centriole is laterally displaced to the side of the nucleus while the HTCA expands under the nucleus, forming what we term the ‘nuclear shelf’. We next show that the proximal centriole-like (PCL) structure is positioned under the nuclear shelf, functioning as a crucial stabilizer of centriole–nucleus attachment. Together, our data indicate that the HTCA is a complex, multi-point attachment site that simultaneously engages the PCL, the centriole and the nucleus to ensure proper head–tail connection during late-stage spermiogenesis.

## Teaser

**Highlighted Article:** A description of two structures at the head–tail attachment site in *Drosophila*, the centriole cap and nuclear shelf, and that the proximal centriole-like structure (PCL) facilitates attachment between the sperm head and tail.

## INTRODUCTION

Fertilization requires a properly formed sperm, which comprises a ‘head’ containing the genetic material and a ‘tail’ that drives sperm motility in species with flagellated sperm. A stable connection between the sperm head and tail is crucial for proper sperm motility and function. Failure to establish or maintain the connection between the head and tail can result in acephalic spermatozoa syndrome in humans, which presents with unstable connections between the sperm head and tail, including bent configurations and completely detached sperm heads, ultimately resulting in infertility (Baccetti et al., 1989; Chemes et al., 1987, 1999; Perotti et al., 1981). Defects at the connection between the sperm head and tail have been linked to infertility across many other species, including in bovines (Blom and Birch-Andersen, 1970), mice (Wang et al., 2022; Zhang et al., 2021) and *Drosophila* (Anderson et al., 2009; Kracklauer et al., 2010; Li et al., 2004; Sitaram et al., 2012; Texada et al., 2008).

Loosely defined, the head-tail coupling apparatus (HTCA, also referred to as the ‘sperm neck’) is a structure within the neck of the sperm that “anchors the flagellum to the sperm head” (Gene-Ontology-Term #0120212; Kierszenbaum et al., 2011). Although the precise arrangement of components within the HTCA varies across species, the overarching theme of HTCAs is to provide a linkage between the nucleus and the tail through centrioles (reviewed in Tapia Contreras and Hoyer-Fender, 2021; Wu et al., 2020). The *Drosophila* HTCA appears to accomplish the task of linking the nucleus to the flagella by directly anchoring the giant centriole (basal body) to the nuclear membrane, although the molecular details of how this is accomplished are scant (Galletta et al., 2020; Tates, 1971; Tokuyasu, 1975a). Numerous proteins have been localized at or near the HTCA in various species. HTCA proteins at the head can be found in the nuclear envelope, including but not limited to the testis-specific SUN domain protein Spag4 and nuclear pore complexes in *Drosophila* (Tates, 1971; Kracklauer et al., 2010). Centrosome proteins, such as CP110, CCDC42, CCDC159 and CEP135 in mice, and CETN1, FAM161A and WDR90 in humans, rabbits and bovines (Khanal et al., 2021; Subbiah et al., 2024; Ge et al., 2024; Tapia Contreras and Hoyer-Fender, 2019), are also found near the HTCA. Finally, various cytosolic proteins localize to the HTCA, including the *Drosophila*-specific gravitaxis protein Yuri Gagarin (Yuri; Kracklauer et al., 2010; Texada et al., 2008), dynein and its adaptor Lis-1 in *Drosophila* (Li et al., 2004; Sitaram et al., 2012), the syntaxin-interacting protein Salto in *Drosophila* (Augiere et al., 2019), the microtubule-binding protein Hook1 in rodents (Kierszenbaum et al., 2011), and the sperm protein PMFBP1 (Zhu et al., 2018) and the linker protein CENTLEIN in mice (Zhang et al., 2021).

Mutations in many proteins localized to the HTCA are linked to head–tail connection defects and infertility. For example, mutations in the testis-specific SUN-domain protein SPAG4L (SUN5) and the sperm-tail associated protein PMFBP1 account for nearly 70% of acephalic spermatozoa cases in humans (Zhang et al., 2021). In *Drosophila*, mutations in Spag4 (the ortholog of mammalian SUN5) and Yuri similarly lead to failed connection at the HTCA and infertility (Kracklauer et al., 2010; Texada et al., 2008). Although many proteins have been localized to the HTCA and linked to infertility, it is unclear how these proteins assemble and function to physically link the sperm head and tail.

Sperm development in *Drosophila* begins with the asymmetric division of germline stem cells, giving rise to cells that undergo multiple divisions, eventually becoming spermatocytes (Fig. S1A) (Demarco et al., 2014; Fuller, 1993). Spermatocytes proceed through meiosis to become spermatids, which then undergo spermiogenesis – a series of dramatic morphological changes in which the axoneme elongates and the nucleus reshapes, ultimately resulting in mature sperm (Fig. S1A–C) (Anderson, 1967; Fabian and Brill, 2012; Shoup, 1967; Stanley et al., 1972; Tates, 1971; Tokuyasu, 1974a,b, 1975a,b, 1972a,b, 1977).

The connection between the nucleus and centriole is first established during early spermiogenesis and must be maintained as the spermatid develops (Fig. S1B,C) (Fabian and Brill, 2012; Tates, 1971). Previous work in *Drosophila* has shown that initial attachment between the nucleus and centriole in Round spermatids is dependent on dynein at the nuclear envelope (Anderson et al., 2009; Li et al., 2004; Sitaram et al., 2012). In addition, restriction of pericentriolar material and microtubule nucleation to the proximal end of the centriole is necessary to ensure proper end-on attachment of the centriole to the nucleus (Galletta et al., 2020). However, less is known about how the nucleus and centriole maintain their connection as spermatids develop and remodel.

Here, we sought to create a high-resolution map of proteins and centriole structures present at the HTCA throughout spermiogenesis as the connection between the nucleus and centriole is established and remodeled. Using structured illumination microscopy, we defined two novel structures at the HTCA during the remodeling that occurs during this period and identified a new role for the proximal centriole-like (PCL) structure, an atypical procentriole, in maintaining centriole positioning relative to the nucleus.

## RESULTS

### The HTCA is remodeled into a ‘centriole cap’ and ‘nuclear shelf’

**Fig. 1.**
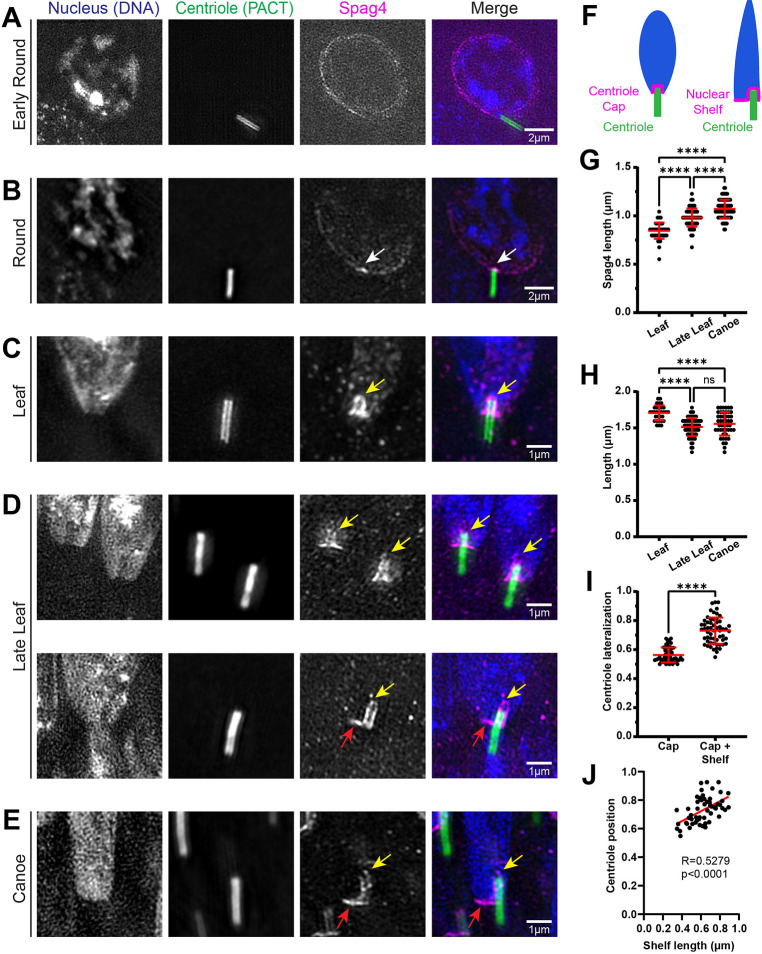
**The HTCA is remodeled into a centriole cap and nuclear shelf.** (A–E) Representative SIM images showing spermatids from wild-type testes during indicated developmental stages. Spermatids were labeled for the nucleus (DAPI, blue), centriole (PACT::GFP, green) and Spag4 (Spag4::6myc, magenta). White arrows denote Spag4 accumulation at centriole docking site. Yellow arrows denote centriole caps. Red arrows denote nuclear shelves. Scale bars are labeled. (F) Cartoons depicting the centriole cap and nuclear shelf. (G) Quantification of Spag4 centriole cap length in Leaf (*n*=50), Late Leaf (*n*=78), and Canoe stage (*n*=85) spermatids. (H) Quantification of centriole length in Leaf (*n*=31), Late Leaf (*n*=72), and Canoe stage (*n*=47) spermatids. (I) Quantification of centriole lateralization index in spermatids with only a centriole cap (*n*=55) and spermatids with a centriole cap and nuclear shelf (*n*=58). (J) Scatter plot fit with a linear regression showing that centriole lateralization increases with shelf length (*n*=56). Error bars are mean±s.d. ns, not significant, ****P≤0.0001 (one-way ANOVA with Tukey's correction for G, H; unpaired two-tailed *t*-test for I).

Although the HTCA is conceptually well understood, how it is established and develops in space and time during spermiogenesis is not well described. Thus, the first goal was to perform structured illumination microscopy (SIM) on known HTCA proteins to define the HTCA structure throughout spermatid development. We selected the testis-specific SUN-domain protein Spag4 and the *Drosophila*-specific gravitaxis protein Yuri, as both localize to the HTCA and mutations in each lead to sperm decapitation (Kracklauer et al., 2010; Texada et al., 2008), indicating a crucial role in maintaining the connection between head and tail. In Early Round spermatids, the nucleus and centriole begin to form an attachment (Fig. S1B,C). At this stage, Spag4 encircled the entire nuclear envelope (Fig. 1A). Shortly after, in Round spermatids, the centriole attached to the nucleus perpendicular to the nuclear surface, or ‘end-on’ (Fig. S1B,C), and Spag4 formed an asymmetrical crescent on one side of the nucleus, centered on the centriole (Fig. 1B) (Kracklauer et al., 2010; Texada et al., 2008). Interestingly, Spag4 accumulated at higher levels in a small region on the nucleus at the centriole attachment point (Fig. 1B, white arrow). In Leaf stage spermatids, where the nucleus begins to elongate (Fig. S1B,C), Spag4 dramatically remodeled into a structure tightly surrounding the proximal end of the centriole (Fig. 1C,F), which begins to invaginate into the nuclear surface (Tokuyasu, 1975a). We termed this structure the ‘centriole cap’. As the nucleus further elongated and narrowed in Late Leaf and Canoe stage spermatids (Fig. S1B,C), the centriole cap lengthened (Fig. 1D,E,G) in a manner corresponding with further invagination. Measurements of centriole length during this process indicated that the centriole did not grow longer (Fig. 1H). Thus, the lengthening of the centriole cap is most likely due to the centriole moving into the nuclear space rather than remaining fixed at a point and growing into the nucleus. Although it has been shown that dynein and microtubules are important in bringing the centriole to the nuclear surface in *Drosophila* (Anderson et al., 2009; Galletta et al., 2020; Li et al., 2004; Sitaram et al., 2012), the mechanism by which the centriole invaginates into the nuclear space is unknown.

By the Canoe stage, we observed nearly half of spermatids with asymmetrical localization of Spag4 (Fig. 1E, red arrow). The asymmetry is a result of an extension of the HTCA to one side of the centriole cap and below the nucleus; we termed this structure the ‘nuclear shelf’. The formation and positioning of the shelf corresponded to the decentralization of the centriole relative to the nucleus (Fig. 1D–F). Measuring the position of the centriole relative to the short axis of the nucleus revealed that centrioles are positioned more laterally when a shelf is present in Leaf through Canoe stages (Fig. 1I). Furthermore, there is a positive correlation between shelf length and the extent of centriole lateralization (Fig. 1J), indicating that the events are coupled. Taken together, we show that the HTCA, as shown by Spag4 (Fig. 1) and Yuri (Fig. S2), forms an asymmetrical crescent that develops into a centriole cap surrounding the proximal end of the centriole as it invaginates into the nuclear membrane. The HTCA is then further remodeled by forming a nuclear shelf at the bottom of the nucleus while the centriole is laterally positioned to one side of the nucleus.

### The centriole adjunct is remodeled as the centriole cap elongates

**Fig. 2.**
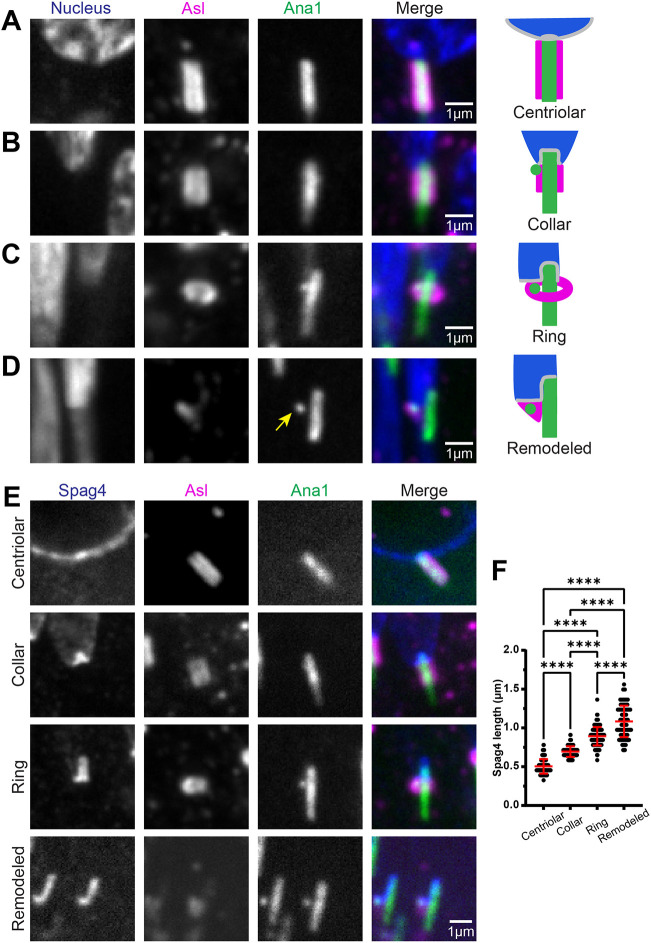
**The centriole adjunct is extensively remodeled as the centriole cap elongates.** (A–D) Representative images showing spermatid centrioles from wild-type testes at indicated stages of CA remodeling (right). Spermatids were labeled for the CA (Asl, magenta) and the centriole and PCL (Ana1::tdTomato, green). Yellow arrow denotes PCL. Scale bars: 1 μm. Cartoons represent different stages of CA remodeling. (E) Representative images showing spermatids from wild-type testes at indicated stages of CA remodeling. Spermatids were labeled for Spag4 (Spag4::6myc, blue), the centriole and PCL (Ana::tdTomato, green), and CA (Asl, magenta). Scale bar: 1 μm. (F) Quantification of Spag4 centriole cap length in spermatids at centriolar (*n*=155), collar (*n*=147), ring (*n*=134), and remodeled (*n*=110) stages of CA remodeling. Error bars are mean±s.d. ****P≤0.0001 (one-way ANOVA with Tukey's correction for F).

Given the extensive remodeling of the HTCA, we sought to investigate other structures associated with spermatid centrioles during spermiogenesis. One such structure is the centriole adjunct (CA), a dense structure of pericentriolar material that surrounds the centriole and helps nucleate a specialized microtubule network called the dense complex (the manchette), which is important for spermatid nuclear shaping in *Drosophila* and mice (Riparbelli et al., 2020; Russell et al., 1991). Electron micrographs show that the CA is positioned near the site of the HTCA in developing *Drosophila* spermatids (Riparbelli et al., 2020; Tokuyasu, 1975a); thus, we hypothesized that the CA associates with, and possibly regulates centriole cap or nuclear shelf formation. To investigate CA dynamics, we immunostained spermatids for the CA component Asterless (Asl, an ortholog of mammalian CEP152), a known regulator of PCM assembly and centriole length (Galletta et al., 2016; Blachon et al., 2008). As previously shown, we found that Asl localized along the entire length of the centriole in Round spermatids (Fig. 2A) (Galletta et al., 2016; Khire et al., 2015). Asl then began to condense into a ‘collar’ and then a ‘ring’ in Leaf and Canoe stage spermatids, respectively (Fig. 2B,C). The transition of the CA to the collar and ring perfectly correlated with the invagination of the centriole into the nucleus (Fig. 2B–D) and formation and elongation of the centriole cap shown by Spag4 (Fig. 2E,F). Finally, Asl was further remodeled asymmetrically in late Canoe spermatids to one side of the centriole, positioned beneath the Spag4 nuclear shelf (Fig. 2D). Thus, we hypothesize that condensation of the CA into a ring exposes the proximal end of the centriole and facilitates centriole invagination into the nucleus and remodeling of the HTCA. Unfortunately, we were unable to directly test whether the CA is important for invagination or important for HTCA formation as *asl* mutants also affect centriole duplication and microtubule nucleation (Blachon et al., 2008; Varmark et al., 2007).

### The PCL is associated with the nuclear shelf

**Fig. 3.**
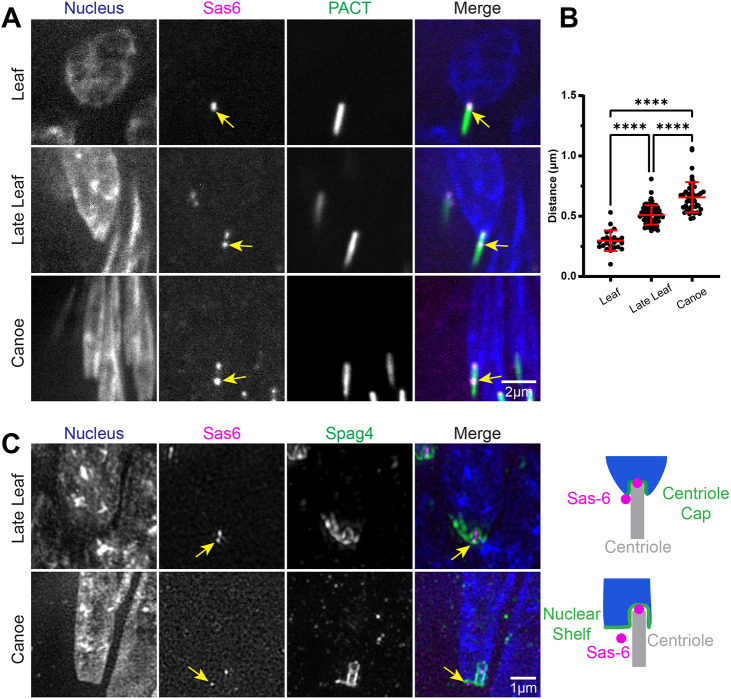
**The PCL is associated with the nuclear shelf during centriole invagination.** (A) Representative images showing spermatids from wild-type testes during indicated developmental stages. Spermatids were labeled for the nucleus (DAPI, blue), centriole (PACT::GFP, green), and Sas6 marking the proximal end of the centriole and the PCL (TagRFP::Sas6, magenta). Scale bar: 2 μm. Yellow arrows denote PCL. (B) Quantification of the distance between the proximal end of the centriole and the PCL in Leaf (*n*=24), Late Leaf (*n*=67) and Canoe (*n*=46) stage spermatids. (C) Representative SIM images from three repeats showing spermatids from wild-type testes during indicated developmental stages. Spermatids were labeled for the nucleus (DAPI, blue), Spag4 (Spag4::6myc, green) and Sas6 marking the proximal end of the centriole and the PCL (TagRFP::Sas6, magenta). Yellow arrows denote PCL. Scale bar: 1 μm. Cartoons depict position of the PCL beneath the centriole cap and nuclear shelf. Error bars are mean±s.d. ****P≤0.0001 (one-way ANOVA with Tukey's correction for B).

As the centriole is positioned laterally on one side of the nucleus, the CA and the centriole cap become asymmetrical (Figs 1D, 2D). Further analysis of the CA in late Canoe stages revealed an Ana1 (centriole marker, ortholog of mammalian CEP295)-positive structure within the CA (Fig. 2D, yellow arrow). This structure is known as the proximal centriole-like (PCL) structure, an atypical procentriole found in insects that serves as a template for daughter centriole formation during the first zygotic divisions (Blachon et al., 2009, 2014). The PCL develops early in spermiogenesis and undergoes significant remodeling as it is incorporated into the mature sperm architecture (Blachon et al., 2009, 2014; Khire et al., 2016). Furthermore, mutations in PCL-associated proteins result in male infertility, immotile sperm and sperm structural abnormalities (Khire et al., 2016). This led us to hypothesize that the PCL is important in defining the nuclear shelf and creating a stable HTCA. To test this hypothesis, we first determined the position of the PCL relative to the nucleus, centriole, centriole cap and nuclear shelf. We utilized transgenic flies expressing TagRFP::Sas6, which labels both the proximal end of the centriole and the PCL (Galletta et al., 2016; Jo et al., 2019). We found that the position of the PCL was nearly indistinguishable from the proximal end of the centriole in Leaf stage spermatids, but became displaced from the proximal end as spermatids transitioned into Late Leaf and Canoe stages (Fig. 3A). We measured the distance between the proximal end of the centriole and the PCL at each stage and found that the Sas6-positive PCL moves further away from the end of the centriole as the centriole invaginates into the nucleus (Fig. 3B). Thus, throughout the centriole invagination process, the PCL was always positioned at the base of the nucleus, eventually settling into a location below the nuclear shelf in Canoe stage spermatids (Fig. 3C) and subsequently at the base of the needle shaped nuclei in mature sperm (Fig. S1B).

Taken together, the PCL is initially formed at the proximal end of the centriole, then it remains associated with the remodeled CA collar and ring in a position below the nucleus where it ultimately becomes a part of the final mature sperm structure.

### The PCL acts as an anchor during centriole invagination

Given that both the CA and PCL are positioned below the nuclear shelf and remodeled during centriole invagination, we hypothesized that the CA or the PCL is necessary for proper assembly or maintenance of the HTCA. Unfortunately, we were unable to directly test this for the CA, but we were able to genetically disrupt the PCL using a mutant for the centriole protein Poc1. The *Drosophila Poc1* gene encodes two protein isoforms, Poc1A, a centriole protein important for centriole elongation, and Poc1B, a centriolar protein that is highly expressed in the testes that exclusively localizes to the PCL (Fig. S3) (Khire et al., 2016).

**Fig. 4.**
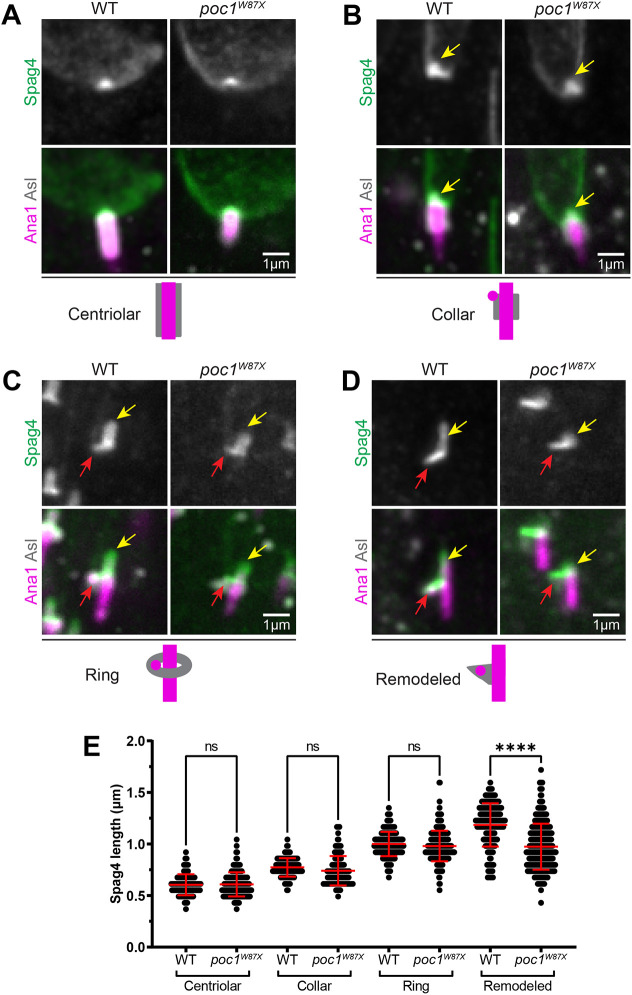
***poc1* mutants have shorter centriole caps following remodeling of the CA.** (A–D) Representative images showing spermatids from wild-type (left) and *poc1^W87X^* mutant (right) testes at indicated stages of CA remodeling. Spermatids were labeled for Spag4 (Spag4::6myc, green), the centriole and PCL (Ana1::tdTomato, magenta), and the CA (Asl, gray). Yellow arrows denote the centriole cap. Red arrows denote the nuclear shelf. Cartoons depict different stages of CA remodeling. Scale bar: 1 μm. Images of individual channels are shown in Fig. S4. (E) Quantification of centriole cap length in wild-type and *poc1^W87X^* mutant spermatids during centriolar (wild-type *n*=86; mutant *n*=148), collar (wildtype *n*=125; mutant *n*=142), ring (wild type *n*=258; mutant *n*=261), and remodeled (wildtype *n*=188; mutant *n*=322) stages of CA remodeling. Error bars are mean±s.d. ns, not significant, ****P≤0.0001 (one-way ANOVA with Tukey's correction for E).

To test the role of the PCL in sperm head–tail connection, we analyzed *poc1^W87X^* mutant flies, which have been previously shown to disrupt the architecture of the PCL (Khire et al., 2016). In Round spermatids, the nucleus and centriole properly made contact to establish the HTCA in both wild type and *poc1* mutants (Fig. 4A; Fig. S4A). Furthermore, in both mutants and controls, the CA was properly localized along the entire centriole length, and Spag4 accurately formed a crescent with the expected accumulation at the site of centriole docking (Fig. 4A; Fig. S4A). In addition, no significant difference in the Spag4 cap length was detected in *poc1* mutants through to Canoe stage spermatids that contained collar and ring shaped CAs (Fig. 4B,C,E; Fig. S4B,C). *poc1* mutants also properly formed Spag4 nuclear shelves and laterally positioned centrioles (Fig. 4C; Fig. S4C), indicating that centriole lateralization and asymmetry of the HTCA is not dependent on the PCL. However, during CA remodeling from a ring to a density beneath the nuclear shelf (Fig. 2D), *poc1* mutant spermatids displayed shorter Spag4 centriole caps (Fig. 4D,E; Fig. S4D). We also tested a hypomorph *poc1* allele (*poc1^c06059^*) that reduces Poc1 expression (Khire et al., 2016), and similarly found a shorter Spag4 cap length in late stage spermatids (Fig. S5A,B). We therefore conclude that the PCL is not required for centriole invagination and lateralization, but is required for maintaining invagination of the centriole in late stages of spermiogenesis.

To ensure that the loss of centriole invagination was due to disruption of the PCL and not due to other effects of *poc1* manipulation, we disrupted the PCL via *plk4* RNAi, as formation of the PCL is dependent on Plk4 (Blachon et al., 2009). Knockdown of Plk4 did not entirely eliminate the PCL in spermatids as evidenced by the presence of a range of Sas6 amounts at the PCL (Fig. S5C). We found that spermatids with reduced Sas6 at the PCL, which we interpret as not fully formed, were not properly invaginated or were completely detached from the sperm nucleus (Fig. S5C,D). Together, our data is consistent with a model whereby Poc1 and the PCL are not necessary for centriole lateralization and establishment of the nuclear shelf, but are crucial for maintaining centriole invagination and anchoring the centriole during later stages.

**Fig. 5.**
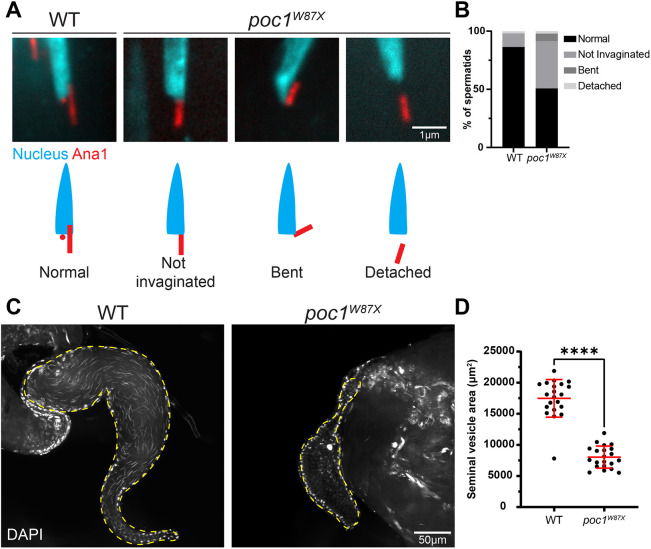
**The PCL is required to maintain centriole invagination in late stages of spermiogenesis.** (A) Representative images showing wild-type (left) and *poc1^W87X^* mutant (right) Canoe stage spermatids with various HTCA phenotypes. Spermatids were labeled for the nucleus (DAPI, cyan) and the centriole and PCL (Ana1::tdTomato, red). Scale bar: 1 μm. Cartoons depict centrioles that are normal, not invaginated, bent, or detached relative to the nucleus. (B) Quantification of wild-type (*n*=367) and *poc1^W87X^* mutant (*n*=597) spermatids with various HTCA phenotypes. (C) Representative images showing wild-type (left) and *poc1^W87X^* mutant (right) seminal vesicles (dashed yellow line). Mature sperm were labeled for the nucleus (DAPI). Scale bar: 50 μm. (D) Quantification of seminal vesicle area in wild-type (*n*=21) and *poc1^W87X^* mutant (*n*=21) testes. Error bars are mean±s.d. ****P≤0.0001 (unpaired two-tailed *t*-test for D).

Given that *poc1* mutant spermatids had significantly shorter Spag4 caps in later stages of development, we hypothesized that centrioles were no longer stably linked to the nucleus. Indeed, we found that 50% of *poc1* mutant centrioles were not properly invaginated, attached at abnormal angles (bent), or completely detached from the nucleus (Fig. 5A,B). *poc1^c06059^* flies similarly had reduced centriole invagination compared to controls, although to a lesser extent than *poc1^W87X^* flies (Fig. S5E,F).

These data suggest that loss of the PCL results in a stepwise deterioration of the centriole–nucleus attachment, starting with a regression from the nucleus, followed by a weakening of the connection, and finally full centriole detachment. Given that the PCL is positioned further from the proximal end of the centriole as the centriole invaginates, it is likely that the PCL remains anchored at the nuclear shelf to act as an additional point of contact to stabilize the centriole during invagination. We finally hypothesized that without proper attachment the ability of the sperm to swim and reach the seminal vesicle would be reduced. Indeed, seminal vesicles from *poc1* mutants were significantly smaller with little to no sperm compared to controls (Fig. 5C,D). This is fully consistent with the previous observation that flies with mutations in *poc1* have immotile sperm and sterility (Khire et al., 2016).

## DISCUSSION

Mature sperm must form a stable connection between the head and tail. This linkage is mediated by the HTCA, which connects the sperm nucleus to the axoneme via the centriole. In *Drosophila*, initial attachment of the centriole is end-on to the nucleus, whereas the final mature sperm form a stable lateral attachment. During the centriole transition from end-on to lateral attachment, the HTCA is remodeled while maintaining the connection between the nucleus and the centriole. Our study used structured illumination microscopy to characterize remodeling of the HTCA and HTCA-associated structures during spermiogenesis. Importantly, we have discovered a new role for the PCL as a structural anchor that maintains and stabilizes sperm tail attachment.

**Fig. 6.**
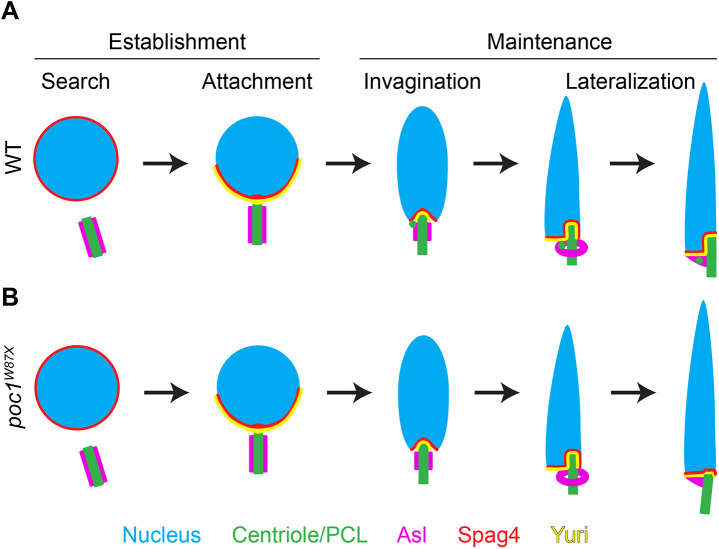
**Model of HTCA establishment and remodeling.** (A) In normal development, the nucleus and centriole use microtubules and dynein (not depicted) during the search phase to initiate their interaction, which leads to the direct centriole–nucleus interaction during the attachment phase. This constitutes establishment of the HTCA. During attachment, spermatid nuclei are Round, the CA is localized along the length of the centriole, and Spag4 and Yuri form a crescent with accumulation at the site of centriole docking. Development proceeds with the maintenance phase, during which the centriole undergoes invagination into the nucleus at Leaf stage spermatids. Spag4 and Yuri form a centriole cap surrounding the invaginating centriole and the CA begins to condense into a collar. As invagination continues into the Canoe stage, the centriole undergoes lateralization during which the centriole moves to one side of the nucleus. On the opposite side, Spag4 and Yuri form a nuclear shelf, with the PCL localized beneath the shelf. The CA further condenses into a ring as the centriole continues to invaginate into the nucleus. In the final stages of lateralization, the CA condenses around the PCL to sit beneath the nuclear shelf. This results in a stable lateral connection between the sperm head and tail. (B) In *poc1* mutants, search, attachment, and invagination all occur, with normal remodeling of the CA and elongation of the nucleus. However, in the final stages of lateralization, centrioles are not anchored by the PCL and lose their invagination, resulting in an unstable attachment between head and tail.

Our data, combined with that from previous studies, reveals a two-phase process of HTCA development (Fig. 6). First is the ‘establishment’ phase, which consists of nuclear ‘search’ by the centriole (stage 1) followed by centriole ‘attachment’ to the nucleus (stage 2). This establishment phase is mediated by dynein and its adaptors Lis-1 and Asunder, and requires restriction of the PCM and microtubules to the proximal end of the centriole to ensure end-on attachment (Anderson et al., 2009; Galletta et al., 2020; Li et al., 2004; Sitaram et al., 2012). Importantly during this time, the CA, as marked by Asl, is positioned along the entire length of the centriole (Fig. 6, magenta). Next is the ‘maintenance’ phase, which consists of centriole ‘invagination’ into the nucleus (stage 3) followed by centriole ‘lateralization’ (stage 4). During centriole invagination, the CA begins to condense and expose the proximal end of the centriole as Spag4 and Yuri form a ‘centriole cap’ around the centriole as it invaginates. As the centriole moves laterally (still remaining associated with the nuclear surface via its proximal and lateral surfaces), Spag4 and Yuri localization expands to the ‘nuclear shelf’ along the bottom of the nucleus. In mammals, the proximal centriole is positioned in a shallow dent in the nucleus known as the implantation fossa (Fawcett, 1975), similar to our observation that the *Drosophila* giant centriole invaginates into the nucleus and the centriole cap. Furthermore, the centriole cap and nuclear shelf might function similarly to the mammalian basal plate, a local thickening on the nuclear envelope at the implantation fossa that functions to link the nucleus and centriole (Fawcett, 1975). Although no such thickening at the nuclear membrane is found in *Drosophila* (Tokuyasu, 1975a), we suspect that the centriole cap and nuclear shelf are collectively a ‘basal plate-like’ structure that serves as a localization site for key HTCA proteins to facilitate head–tail linkage.

Concomitantly with invagination and lateralization of the centriole is the formation and movement of the PCL and restructuring of the CA. Presumably, these events are coordinated to ensure that the final positions of the CA and PCL are below the nuclear shelf. One possibility is that the PCL actively moves along the centriole, dragging the nuclear envelope with it to surround the centriole during invagination. This led us to hypothesize that the PCL would be crucial for invagination and/or lateralization of the centriole in the nucleus during HTCA formation. We therefore investigated *poc1* mutants, which have abnormal PCL structures. Interestingly, spermatids in these mutants are able to progress through centriole invagination and lateralization, indicating that the PCL does not play a role in these early steps of HTCA formation and likely does not drag the nuclear envelope around the centriole. However, these spermatids are unable to maintain centriole invagination into the nucleus in late stages of spermiogenesis, indicating a role for the PCL in stabilizing the final head–tail connection. This is consistent with the PCL being anchored below the nuclear shelf as the centriole invaginates into the nuclear space. We propose that the HTCA forms a multi-point, L-shaped attachment surface that links the centriole, PCL and the nucleus. Such an expanded attachment site might be crucial for resisting forces of the swimming tail and ensuring its stable connection to the head. This is consistent with our finding that *poc1* mutant flies have little to no sperm in their seminal vesicles, as sperm must be able to swim in order to reach the seminal vesicle. In humans, sperm with abnormal morphology have reduced expression of *POC1B* mRNA (Platts et al., 2007), and abnormal levels of POC1B at the centrioles have been found in individuals with unexplained infertility (Turner et al., 2021). Furthermore, the atypical centriole in human and bovine sperm has an enrichment of POC1B (Khanal et al., 2021; Fishman et al., 2018). Interestingly, at least in bovine sperm, this atypical centriole appears to be important in creating a specialized coupling between the head and tail to control movement (Khanal et al., 2021). Together, these observations all support the hypothesis that atypical centrioles like the PCL are necessary for proper sperm motility and fertility across species.

One interesting aspect of our study was the relationship between the CA and the nucleus. Several previous studies have shown that the CA at some point forms a ring structure (Blachon et al., 2008; Galletta et al., 2016; Tates, 1971); however, not much more was known. By careful timing and correlation with centriole dynamics, we observed that the CA localized along the entire centriole through the centriole ‘attachment’ phase. Then, the CA began to remodel by moving toward the longitudinal center of the centriole, exposing both the distal and proximal ends. The mechanism by which the CA condenses into a focused ring and then a density under the nucleus is completely unknown. However, the CA remodeling could involve a phase transition centered around the PCL as it moves from its initial position at the proximal end of the centriole to its final position in the middle of the centriole, just below the nuclear shelf. It is also possible that PCL dynamics and CA remodeling occur independently of one another. Future work to test the role of the PCL in forming the CA will require mutations that would completely eliminate the PCL rather than disrupt its structure, as seen in *poc1* and *sas6* mutations. If such a mutant can be found, it will allow one to probe the relationship between the PCL, CA remodeling and exposure of the proximal end of the centriole, as well as between centriole exposure and invagination. In addition, mutations that disrupt CA formation that do not affect other aspects of spermiogenesis will be important to identify. Such CA mutations will allow one to test several hypotheses, such as the possible role of CA condensation upstream of PCL movement along the centriole, or to investigate whether PCL movement without the CA is sufficient for driving proper centriole invagination.

Finally, in our study we revisit the proteins Spag4 and Yuri, which have been shown to be crucial for the HTCA and male fertility and are hypothesized to function in the same pathway (Kracklauer et al., 2010; Texada et al., 2008). In support of this, we found that they have identical localizations and dynamics during HTCA remodeling – they both form a centriole cap and nuclear shelf. However, it is unclear precisely how Spag4 and Yuri function, independently or in unison. A previous study found that Spag4 and Yuri depend on each other to properly localize to the HTCA (Kracklauer et al., 2010). Similarly, a recent study in mice found that SUN5 and the cytoplasmic protein Septin12 colocalize at the HTCA and that accumulation of SUN5 at the implantation fossa is dependent on Septin12, whereas Septin12 localization depends upon SUN5 (Zhang et al., 2024). How cytoplasmic proteins like Yuri and Septin12 link to Spag4 and SUN5, which are presumed to be inner-nuclear membrane proteins, is unclear. Future work will focus on their mechanistic roles and the identification of proteins that might link Spag4 with Yuri. More broadly, a more complete understanding of the molecular composition of the HTCA will be required to fully investigate how the HTCA forms and accomplishes the crucial task of head–tail connection.

## MATERIALS AND METHODS

### Flies

*D. melanogaster* were maintained and crosses were performed at 25°C. UAS-Ana1::tdTomato expresses Ana1 at endogenous levels without the use of Gal4 (Blachon et al., 2008). We used the following strains: *y,w*, *UAS-Ana1::tdTomato, ubi-PACT::GFP* (Galletta et al., 2020; Martinez-Campos et al., 2004)*, Spag4::6myc* (Bloomington Drosophila Stock Center, Bloomington, IN, USA, #29981, #29980), *TagRFP::Sas6* (Galletta et al., 2020), *poc1A::GFP* (Tomer Avidor-Reiss, Department of Biological Sciences, University of Toledo, USA), *poc1B::GFP* (Tomer Avidor-Reiss), *poc1^W87X^* (Tomer Avidor-Reiss), *poc1^c06059^* (Tomer Avidor-Reiss), and *plk4* RNAi (Bloomington Drosophila Stock Center, #57221).

### Testis fixation and immunofluorescence

Testes from 1–3-day-old males were dissected in Schneider's medium with antibiotic-antimycotic and fixed in 9% paraformaldehyde (PFA) at room temperature (RT) for 15–20 min. Testes were washed in PBS with 0.3% Triton X-100 (PBST) then blocked for 2–6 h at RT in PBST with 5% normal goat serum. Samples were incubated in primary antibody in blocking solution at 4°C overnight, washed three times for 10 min in PBST, then left in secondary antibody in blocking solution for 6–8 h at RT. Following three 10 min washes in PBST, samples were mounted in AquaPolymount (Polysciences, Inc., Warrington, PA, USA) for confocal imaging or Vectashield (Vector Labs, Newark, CA, USA) for SIM. For visualization of Yuri, pharate adults were dissected as above and fixed in 3% PFA at RT for 10 min. Staining was then performed as above. A complete antibody list and concentrations used can be found in Table S1.

### Confocal imaging

Confocal images were primarily acquired using a Nikon Eclipse Ti2 (Nikon Instruments, Melville, NY, USA) with a Yokogawa CSU-W1 spinning disk confocal head (Yokogawa, Life Science, Sugar Land, TX, USA) equipped with a Prime BSI cMOS camera (Teledyne Photometrics, Tucson, AZ, USA) and Nikon Elements software (Nikon Instruments). Spermatids were imaged using a 100×/1.35 NA silicone immersion oil objective or 100×TIRF/1.49 NA oil immersion objective. Some images were acquired using a Nikon Eclipse Ti2 (Nikon Instruments) with a CSU-22 spinning disk confocal head (Visitech International, Allen, TX, USA) equipped with an ORCA-Flash 4.0 CMOS camera (Hamamatsu Photonics, Hamamatsu City, Shizuoka, Japan) and MetaMorph software (Molecular Devices, San Jose, CA, USA) or a Nikon Eclipse Ti2 (Nikon Instruments) with a Yokogawa CSU-X1 spinning disk confocal head (Yokogawa, Life Science) equipped with an OCRA-Flash 4.0 CMOS camera (Hamamatsu Photonics) and Nikon Elements software (Nikon Instruments). Spermatids were imaged using a 100×TIRF/1.49 NA oil immersion objective; seminal vesicles were imaged using a 40×/1.30 NA oil immersion objective. 405, 488, 561, and 641 nm laser lines were used on all systems. All images were analyzed and processed in FIJI (ImageJ, National Institutes of Health, Bethesda, MD, USA).

### Structured illumination microscopy

SIM images were acquired using an OMX4 (GE Healthcare, Chicago, IL, USA) with DeltaVision Immersion Oil 1.514 and a 60×1.42 NA oil immersion objective (Olympus, Center Valley, PA, USA). To ensure successful reconstruction, laser settings for each channel were adjusted to produce a 1000 unit difference between the minimum and maximum intensity. Images were reconstructed with channel specific OTFs and aligned using the softWoRx 3D SIM image reconstruction software (Cytiva, Marlborough, MA, USA).

### Spermatid staging

To stage spermatids based on the shape of the nucleus, a line was drawn through the widest part of the nucleus. Spermatid cysts with nuclei wider than 3 µm were considered ‘Leaf’, nuclei between 2 and 3 µm were considered ‘Late Leaf’, and nuclei narrower than 2 µm were considered ‘Canoe’.

Asl labeling was used to stage spermatids based on the shape of the CA. Spermatids where Asl coated the length of the centriole were considered ‘centriolar’, spermatids where Asl formed a collar over half the centriole were considered ‘collar’, spermatids where Asl formed a ring were considered ‘ring’, and spermatids where Asl was condensed to one side of the centriole were considered ‘remodeled’.

### Analysis of Spag4 length

Only spermatids where a Spag4 Cap was visible and where the centriole was in line with the nucleus were used. A line scan of the Spag4 signal was taken by drawing a 10-pixel-wide by 3-µm-long line centered on the middle of the cap in Fiji software. Background was eliminated by subtracting the average of the first five and last five intensity values from each value. Each intensity value was then normalized to the maximum intensity and the number of pixels which were more than 25% of the maximum was determined and then converted into micrometers.

### Analysis of centriole length

Only spermatids where the centriole was parallel to the imaging plane were used. A line scan of the centriole (as labeled by PACT) was taken by drawing a 5-pixel-wide by 3-µm-long line centered on the middle of the centriole in Fiji software. Background was eliminated by subtracting the average of the first five and last five intensity values from each value. Each intensity value was then normalized to the maximum intensity and the number of pixels which were more than 50% of the maximum was calculated. This was then converted into micrometers to determine centriole length.

### Analysis of centriole lateralization

Spermatids were binned by whether they had only a cap or both a cap and shelf. A 5-pixel-wide line was drawn across the width of the nucleus at the top of the cap, perpendicular to the centriole in Fiji software. In cases where there was a shelf, the beginning of the line was always set at the edge of the nucleus with the shelf. This line was moved down to the bottom of the nucleus and a line scan was taken through the centriole (PACT signal). The position of the maximum intensity of the centriole signal along this line was divided by the length of the line. If the centriole was perfectly centered in the nucleus, this would give a value of 0.5. If the centriole was positioned completely laterally, this would give a value of ∼1.

To determine the correlation between centriole lateralization and shelf length, only spermatids with a visible shelf were selected. The length of the shelf was determined by drawing a line from the end of the shelf to the edge of the centriole. The centriole lateralization was calculated as above and graphed relative to the shelf length. Graphpad Prism was used to determine the Pearson correlation coefficient.

### Analysis of PCL movement

Only spermatids where the PCL (as labeled by Sas6) was in line with the centriole were used. A line scan of the Sas6 signal was taken by drawing a 5-pixel-wide by 2-µm-long line through the Sas6 signal along the centriole and centered between the proximal end of the centriole and the PCL in Fiji software. Background was eliminated by subtracting the average of the first and last intensity values from each value. A sum of two Gaussians was performed in Graphpad Prism and the peak-to-peak distance was calculated to determine the distance between the Sas6 signal from the proximal end of the centriole to the Sas6 from the PCL.

### Analysis of centriole invagination

Only spermatids where the centriole and nucleus could be identified were used. Centrioles that overlapped with the DAPI signal were considered ‘normal’. Centrioles that were attached to and in line with the nucleus but did not overlap with the DAPI signal were considered ‘not invaginated’. Centrioles that were not invaginated but were found at an irregular angle relative to the nucleus were considered ‘bent’. Centrioles that were not in contact with the nucleus were considered ‘detached’.

### Analysis of Sas6 fluorescence at the PCL

Only spermatids where the attachment of the centriole could be accurately assessed were used. A circular region of interest (ROI) was drawn around the Sas6 signal at both the proximal end of the centriole and at the PCL in the *Z*-slice with the brightest signal. An ROI of the same size was taken from the adjacent background in the same slice and subtracted. Fluorescence of Sas6 at the PCL was divided by the fluorescence at the proximal centriole end to generate a ratio. Spermatids were then binned by centriole invagination into one of the following categories: normal, not invaginated or detached.

### Statistics

For comparisons between two groups, unpaired two-tailed *t-*tests were used. Except where noted, one-way ANOVA with Tukey's correction was used for comparisons between three or more groups. Sample sizes are denoted in the figure legends. All statistics were performed using GraphPad Prism 10.0.3 (GraphPad Software, Boston, MA, USA). Error bars represent the mean±s.d. for each graph.

## Supplementary Material



10.1242/joces.262311_sup1 Supplementary information
