# Supplementary material for: The proximal centriole-like structure maintains nucleus–centriole architecture in sperm
Source: J Cell Sci. 2024 Sep 6;137(17):jcs262311. doi: 10.1242/jcs.262311 (PMC11423811; doi:10.1242/jcs.262311)
Supplement: Supplementary information [file joces-137-262311-s1.pdf]

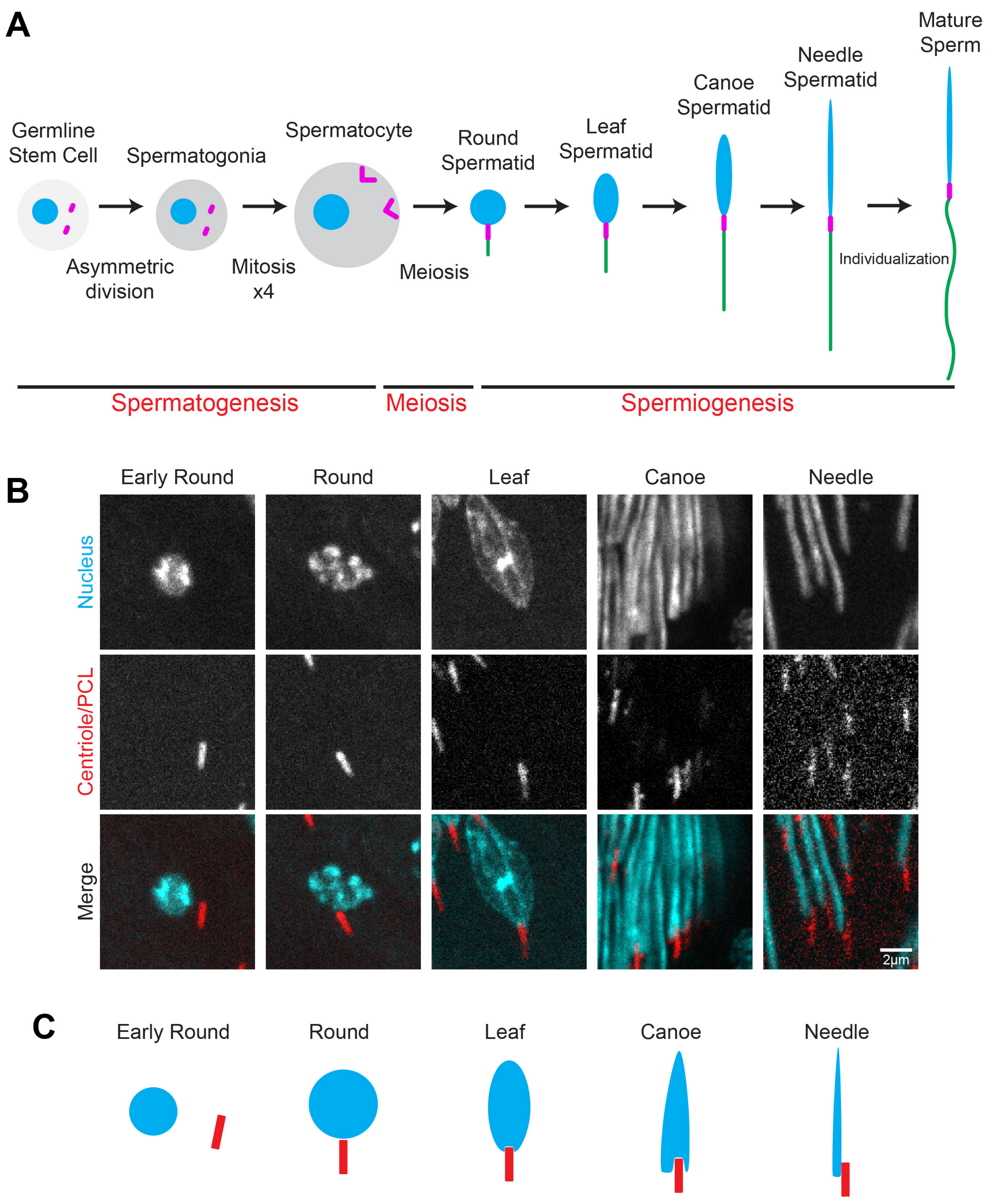

Figure S1. Buglak et al

Journal of Cell Science • Supplementary information

Fig. S1. The centriole and nucleus come together to form a lateral attachment in mature spermatids.

(A) Cartoon depicting sperm development in *Drosophila*. In spermatogenesis, germline stem cells undergo an asymmetric division to generate gonioblasts (not depicted). These cells undergo 4 rounds of mitosis, when they are termed spermatogonia. Following these divisions, these cells become spermatocytes. Spermatocytes go through meiosis is to become spermatids. Spermatids undergo a series of dramatic morphological changes in which the nucleus (head) reshapes and the tail elongates in a process called spermiogenesis. **(B)** Representative images of spermatids at the indicated stages of spermiogenesis, beginning with Early Round spermatids through to Needle stage spermatids. Spermatids were labeled for the nucleus (DAPI, cyan) and the centriole/PCL (Ana1::tdTomato, red). Scale bar, 2µm. **(C)** Cartoons depicting the nucleus and centriole at each stage of spermiogenesis.

Journal of Cell Science • Supplementary information

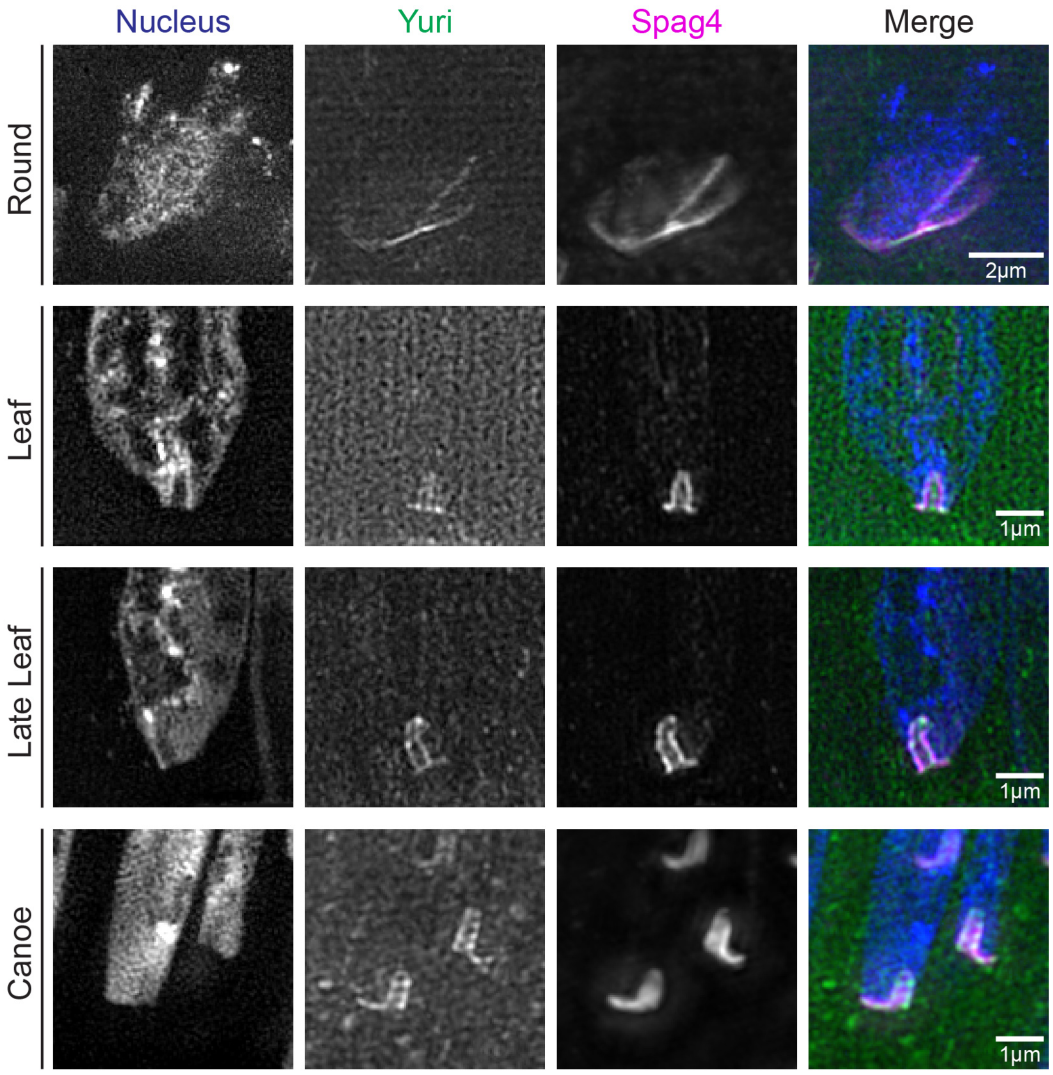

Figure S2. Buglak et al

Fig. S2. Yuri colocalizes with Spag4 at the Centriole Cap and Nuclear Shelf Representative SIM images showing spermatids from wildtype testes during indicated developmental stages. Spermatids were labeled for the nucleus (DAPI, blue), Yuri (green), and Spag4 (Spag4::6myc, magenta). Scale bars, 2 µm (Round), 1 µm (Leaf, Late Leaf, and Canoe).

Journal of Cell Science • Supplementary information

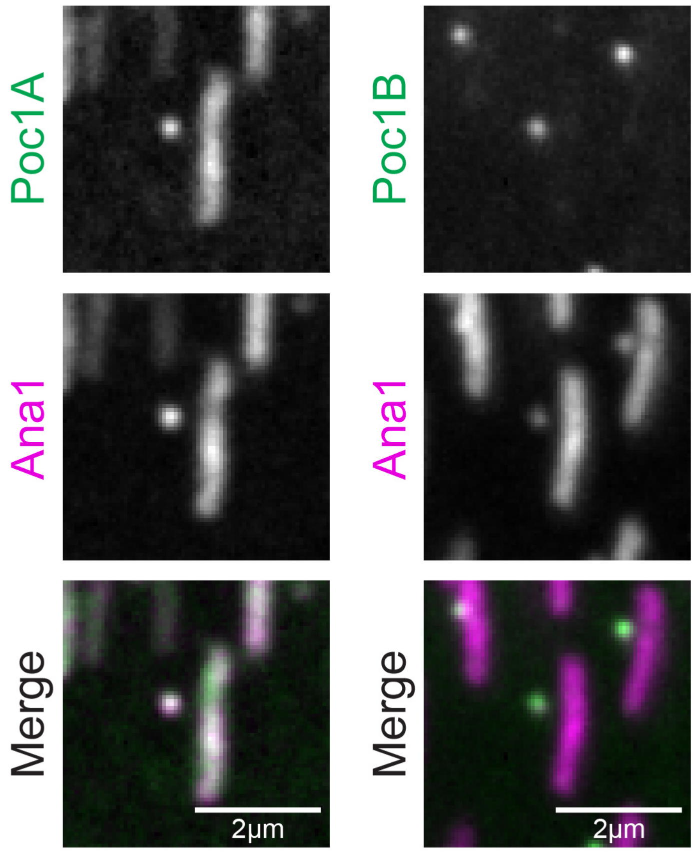

Figure S3. Buglak et al

Fig. S3. Poc1A and Poc1B localize to the PCL.

Representative images of centrioles from Canoe stage spermatids. Spermatids were labeled for the centriole/PCL (Ana1::tdTomato, magenta) and Poc1A (Poc1A::GFP, green, left) or Poc1B (Poc1B::GFP, green, right). Scale bar, 2 µm.

Journal of Cell Science • Supplementary information

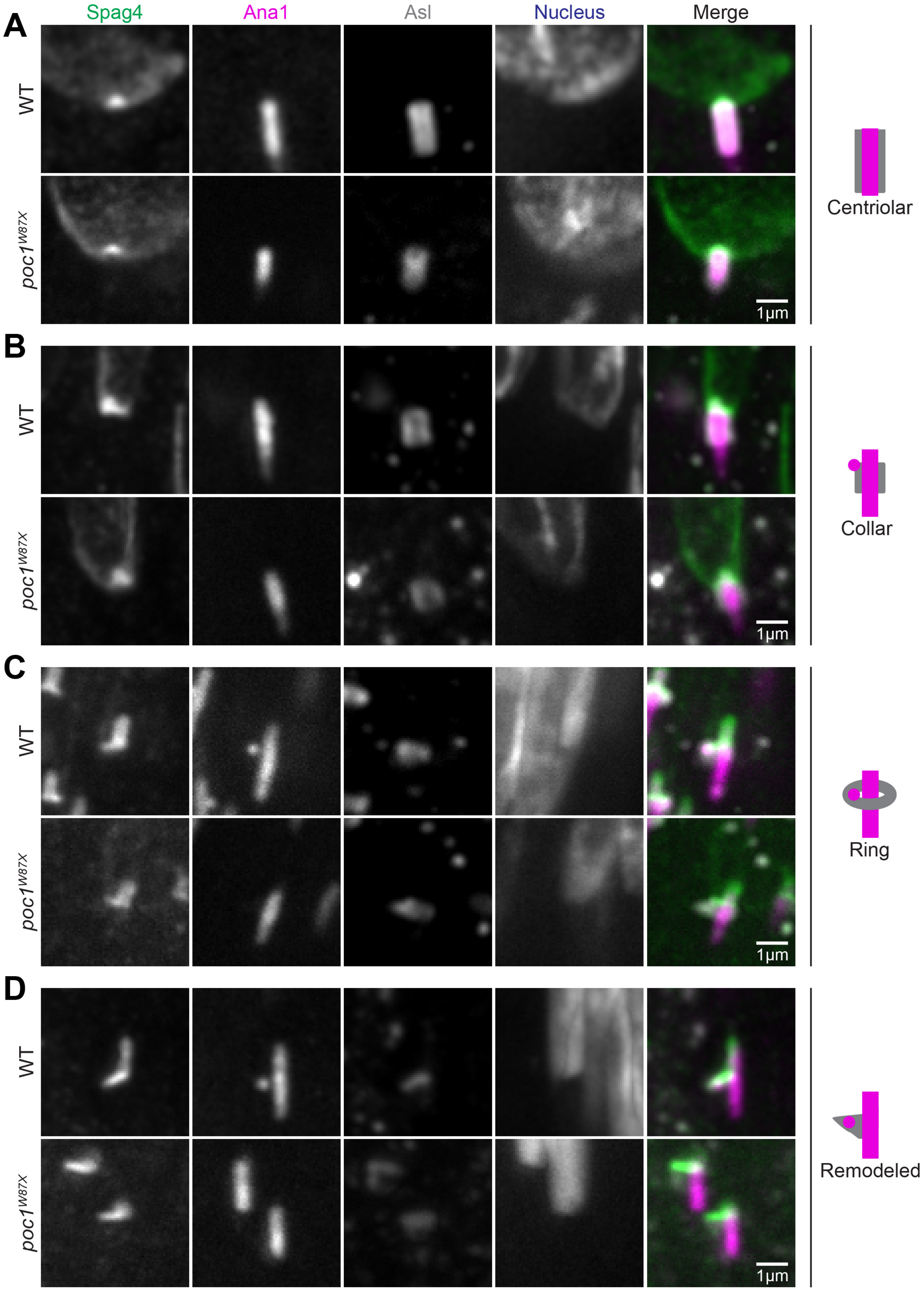

Figure S4

Journal of Cell Science • Supplementary information

Fig. S4. ***poc1*** **mutants have shorter Centriole Caps following remodeling of the CA.**

(A-D) Individual channels for images of wildtype and *poc1* mutant spermatids shown in **Figure 4** at indicated stages of CA remodeling. Spermatids were labeled for the nucleus (DAPI, blue), Spag4 (Spag4::6myc, green), centriole (Ana1::tdTomato, magenta), and CA (Asl, gray). Scale bar, 1µm.

Journal of Cell Science • Supplementary information

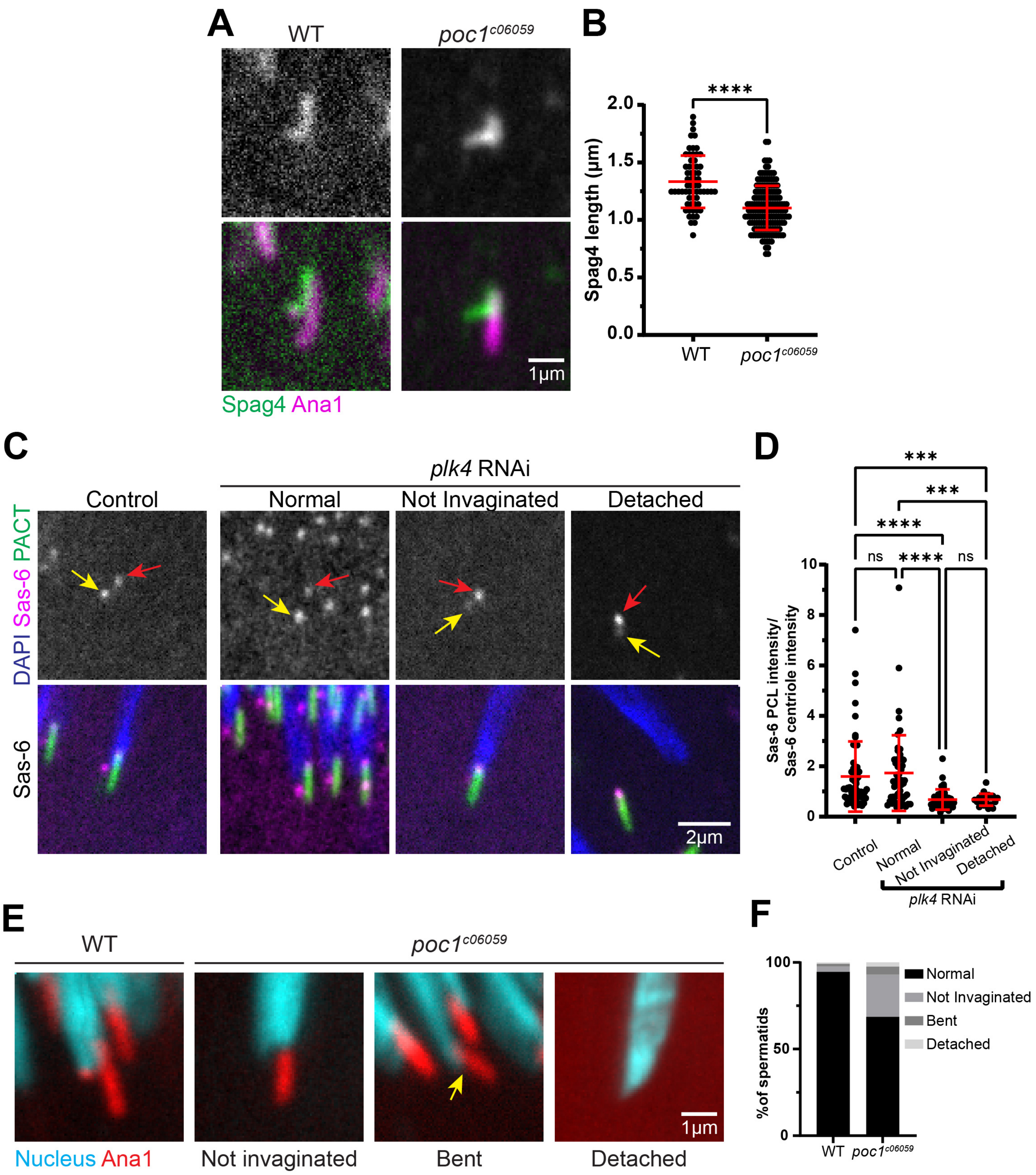

Figure S5. Buglak et al

Journal of Cell Science • Supplementary information

Fig. S5. The PCL is required for proper centriole insertion.

(A) Representative images showing spermatids from wildtype (left) and *poc1*^{c06059} mutant (right) testes at final stages of CA remodeling. Spermatids were labeled with Spag4 (Spag4::6myc, green) and the centriole/PCL (Ana1::tdTomato). Scale bar, 1µm. **(B)** Quantification of Centriole Cap length in wildtype and *poc1*^{c06059} mutant spermatids during the remodeled (wildtype n=63; mutant n=163) stage of CA remodeling. (****) p≤0.0001. **(C)** Representative images showing Canoe stage spermatids from control (left) and *plk4* RNAi (right) with various HTCA phenotypes. Spermatids were labeled with the nucleus (DAPI, blue), centriole (PACT::GFP, green), and Sas-6 (TagRFP::Sas6, magenta). Yellow arrows denote Sas-6 signal at the PCL. Red arrows denote Sas-6 signal at the proximal end of the centriole. Scale bar, 2µm. **(D)** Quantification of Sas-6 signal at the PCL relative to Sas-6 signal at the proximal end of the centriole in control (n=59), *plk4* RNAi normal (n=52), *plk4* RNAi not inserted (n=38), and *plk4* RNAi detached (n=22) Canoe stage spermatids. ns=not significant, (***) p≤0.001, (****) p≤0.0001 by Kruskal-Wallis non-parametric test. **(E)** Representative images showing a wildtype (left) spermatid with normal centriole insertion and a *poc1*^{c06059} mutant (right) spermatid with a centriole that is not inserted. Spermatids were labeled with the nucleus (DAPI, cyan) and the centriole/PCL (Ana1::tdTomato, red). Yellow arrow denotes "bent" spermatid. Scale bar, 1µm. **(F)** Quantification of wildtype (n=128) and *poc1*^{c06059} mutant (n=213) spermatids with various HTCA phenotypes.

Journal of Cell Science • Supplementary information

Table S1. Related to Testes fixation and immunofluorescence section

Antibody/Stain	Type	Source	Catalog #	Dilution
Mouse anti-9E-10 c-myc	Primary	DHSB, Iowa City, IA, USA	9E 10-c	1:1,000
Guinea pig anti-Asl	Primary	(Klebba et al., 2013)		1:10,000
Chicken anti-Yuri 4012	Primary	(Texada et al., 2008)		1:100
AlexaFluor488 conjugated	Secondary	Thermo Fisher Scientific, Waltham, MA, USA		1:1,000
AlexaFluor568 conjugated	Secondary	Thermo Fisher Scientific		1:1,000
AlexaFluor647 conjugated	Secondary	Thermo Fisher Scientific		1:1,000
DAPI	N/A	Thermo Fisher Scientific	D1306	1:100 – 1:1,000
Hoescht 33342	N/A	Life Technologies, Carlsbad, CA, USA	H3570	1:100

Reference

Klebba, J. E., Buster, D. W., Nguyen, A. L., Swatkoski, S., Gucek, M., Rusan, N. M. and Rogers, G. C. (2013). Polo-like kinase 4 autodestructs by generating its Slimb-binding phosphodegron. *Curr. Biol.* **23**, 2255-2261. doi:10.1016/j.cub.2013.09.019
