# Supplementary material for: The proximal centriole-like structure maintains nucleus–centriole architecture in sperm
Source: J Cell Sci. 2024 Sep 6;137(17):jcs262311. doi: 10.1242/jcs.262311 (PMC11423811; doi:10.1242/jcs.262311)
Supplement: Supplementary file 1 [file joces-137-262311_review_history.pdf]

The proximal centriole-like structure maintains nucleus-centriole architecture in sperm

Danielle B. Buglak, Kathleen H. M. Holmes, Brian J. Galletta and Nasser M. Rusan
DOI: 10.1242/jcs.262311

Editor: Renata Basto

Review timeline
Original submission: 30 May 2024
Editorial decision: 5 July 2024
First revision received: 19 July 2024
Accepted: 25 July 2024

Original submission

First decision letter

MS ID#: JOCES/2024/262311

MS TITLE: The Proximal Centriole-Like Structure Anchors the Centriole to the Sperm Nucleus

AUTHORS: Danielle B Buglak, Kathleen H Holmes, Brian J Galletta, and Nasser M Rusan
ARTICLE TYPE: Research Article

We have now reached a decision on the above manuscript.

To see the reviewers' reports and a copy of this decision letter, please go to: https://submit-jcs.biologists.org and click on the 'Manuscripts with Decisions' queue in the Author Area. (Corresponding author only has access to reviews.)

As you will see, the reviewers gave favourable reports but raised some critical points that will require amendments to your manuscript. I hope that you will be able to carry these out because I would like to be able to accept your paper, depending on further comments from reviewers.

Please ensure that you clearly highlight all changes made in the revised manuscript. Please avoid using 'Tracked changes' in Word files as these are lost in PDF conversion.

I should be grateful if you would also provide a point-by-point response detailing how you have dealt with the points raised by the reviewers in the 'Response to Reviewers' box. Please attend to all of the reviewers' comments. If you do not agree with any of their criticisms or suggestions please explain clearly why this is so.

Reviewer 1

Advance summary and potential significance to field

Unlike somatic cells, sperm cells evolve atypical centrioles rapidly, and understanding the role of this atypical centriole is both timely and a priority if we are to better understand how sperm function. This paper provides an important and significant understanding of the role of the PCL, an atypical centriole found in insects in sperm head-neck architecture, and is, therefore excellently suitable for publication after the minor corrections described below.

© 2024. Published by The Company of Biologists under the terms of the Creative Commons Attribution License (https://creativecommons.org/licenses/by/4.0/).

The paper shows that the fly sperm nucleus base is remodeled into a 'Centriole Cap' and 'Nuclear Shelf,' structures that resemble the basal plate in mammals.

It also shows that the PCM (aka insects' centriole adjunct) remodeling is a parallel event, possibly allowing the giant centriole to penetrate the nucleus base. The paper also shows that the PCL is associated with the nuclear shelf moving along the GC as the GC invaginate the nucleus. Affecting the PCL by mutating POC1B or PLk4 RNAi resulted in the loss of maintaining GC invagination; however, the sperm neck maintained attached to the head.

An important emerging picture gained by this paper is that centriole remodeling is coupled to sperm head-neck coupling, paving the way to study if this link is conserved in other animal gropes, such as mammals. Interestingly, the PCL defect and POC1 did not break the connection of the head to the neck but affected the presence of neck/head attachment architecture.

Comments for the author

The title may be better changed from "The Proximal Centriole-Like Structure Anchors the Centriole to the Sperm Nucleus" to "The Proximal Centriole-Like Structure Maintains the Architecture of the Sperm Centriole—Nucleus Anchoring," as it is not clear to me that PCL's direct role in anchoring the Centriole to the Sperm Nucleus is established.

The term HTCA is commonly used in mammal sperm, but the term sperm neck is more commonly used in fly research. Please add that HTCA is also known as sperm neck.

To help a more general audience understand the paper, it will help to add that Spag4 is also known as SUN5 at the first time the protein is presented in the text. Also, indicate the mammalian orthologs of Drosophila proteins, such as Yuri Gagarin, and otherwise indicate if they are insect specific.

Please indicate the model system used to make a particular finding. For example – are these mice or fly: "Previous work discovered that initial attachment between the nucleus and centriole in Round spermatids is dependent on dynein at the nuclear envelope (Anderson 85 et al., 2009; Li et al., 2004; Sitaram et al., 2012)." Add that information throughout the paper.

The "Centriole Cap" and "Nuclear Shelf" are reminiscent of the basal plate, and I wonder why the author does not prefer the term collectively as "basal plate-like."

You may want to address a recent paper in the current paper: Yunfei Zhang, Gang Liu, Lihua Huang, Xiyi He, Yuyan Su, Xinmin Nie, Zenghui Mao, Xiaowei Xing, SUN5 interacts with nuclear membrane LaminB1 and cytoskeletal GTPase Septin12 mediating the sperm head-and-tail junction, Molecular Human Reproduction, 2024;, gaae022, https://doi.org/10.1093/molehr/gaae022.

Reviewer 2

Advance summary and potential significance to field

The basal body-spermatid nucleus interaction is a vital aspect of animal spermiogenesis., since the correct positioning of the axoneme is directly correlated to its function during fertilization. Anormal basal body-spermatid nucleus interaction has been indeed correlated to infertility problems in humans.

However, how the basal body interacts with the nucleus during spermiogenesis progress has not been until now satisfying clarified.

Comments for the author

The main aim of the manuscript entitled "The proximal centriole-like structure anchors the centriole to the sperm nucleus" is to investigate the dynamics of the basal body-nucleus

© 2024. Published by The Company of Biologists under the terms of the Creative Commons Attribution License (https://creativecommons.org/licenses/by/4.0/).

interactions during the process of spermiogenesis in Drosophila melanogaster. The AA addressed this aspect by examining the localization on the centriole adjunct of specific proteins, i.e. Spag4, Yuri Poc1, to clarify the role of these actors in the dynamics of the centriole adjunct and basal body anchoring and stabilization to the basal side of the sperm nucleus. In my opinion the present manuscript is interesting and deserves publication, since the problem addressed is a general aspect of the spermiogenesis that has so far been poorly understood. I have only a few marginal questions that could be easily addressed by the AA.

Minor comments

l.35-36. “Fertilization requires a properly formed sperm, which is comprised of a ‘head’ containing the genetic material and a ‘tail’ that drives sperm motility”. This is an general condition, but there are several examples (insects, nematodes, etc.) in which the sperm are aflagellate and lack the axoneme.

It would be useful for readers if the AA describes the dynamics of the PCL. In l.163 the AA claim “This structure is known as the proximal centriole-like (PCL) structure, an atypical procentriole found in spermatids that serves as a template for daughter centriole formation during the first zygotic divisions. I think that the AA refer to the publish data, but it is unclear for me why the PCL is not found as distinct structure in mature sperm. The PCL is visible as incomplete procentriole during spermatid elongation of several insect species but it is no longer visible as a structured organelle in mature sperm.

L. 119. “Thus the lengthening of the Centriole Cap is due to the centriole moving into the nuclear space rather than remaining fixed at a point and growing into the nucleus”. Are there published data explaining the centriole movement?

I agree with the AA that Spag4 have an internal localization with respect to Yuri that is a cytoplasmic proteins, but from the pictures it seems that the proteins colocalize on the nuclear envelope. It is very interesting! Please explain.

Fig. 2D. The Asl staining is very weak and I can see two small Asl clusters close to the nucleus. Please explain.

l. 206-208. “We therefore conclude that the PCL is not required for centriole invagination and lateralization, but is required for maintaining invagination of the centriole in late stages of spermiogenesis.” It is unclear the relationship between PCL movement and basal body stabilization to the nucleus. Please, could the AA better explain?

l. 286-289. “However, the CA remodelling could involve a phase transition centered around the PCL as it moves from its initial position at the proximal end of the centriole to its final position in the middle of the centriole, just below the Nuclear Shelf”. I understand the hypothesis of the AA, but how the AA can exclude a passive movement of the PCL following CA remodelling? Are there data on PCL dynamics in mutants for proteins that affect the CA?

Fig 1S B,C The lateral position of the basal body at the needle stage is not so evident in immunofluorescence pictures.

<u>Reviewer 3</u>

Advance summary and potential significance to field

In this new and original research Buglak and colleagues investigated the mechanism by which the Head-Tail Coupling Apparatus (HTCA) secures the axoneme to the nucleus in elongating Drosophila spermatids. Using two prominent HTCA components, Spag4 and Yuri as markers they looked at the formation and stabilization of HTCA during spermatogenesis and they did a nice morphological characterization by super-resolution microscopy.

© 2024. Published by The Company of Biologists under the terms of the Creative Commons Attribution License (https://creativecommons.org/licenses/by/4.0/).

Seeking for mechanism involved in remodeling of HTCA, they demonstrated a correlation between condensation of the centriole adjunct (CA, marked with Asterless antibody) and the invagination of the centriole coupled with HTCA remodeling. However, as authors correctly admit, it is difficult to reveal the direct mechanism with classical genetic tools as asterless mutants affect centriole duplication and microtubule nucleation as well.

Moreover, using Sas6 and Ana1 as markers, they investigated the role of procentriole-like structure (PCL) in the context of HTCA formation. The mechanistic glimpse comes from poc1B mutant lacking proper PCL, and the conclusion is that PCL is required in stabilization of the final head-tail connection.

As a conclusion, we see here a work which provides new and original scientific knowledge, lays a good foundation for further mechanistic HTCA research, and it has a potential to be interesting for a wider scientific audience, including broad research areas working on male fertility. It already gives an explanation why abnormal level of POC1B could cause sperms with abnormal morphology and unexplained infertility in humans.

Comments for the author

In the abstract at line 24 there is statement „high-resolution map of proteins and structures at the HTCA". This line promises to much, generates and expectation which is not satisfied by the article itself. Based on this the reader somehow expect proteomics done in an attempt to find all HTCA components strengthen by protein-protein interaction maps and they localization in the mutants of each other. Given the fact that only two HTCA proteins, Spag4 and Yuri were investigated, I would soften this sentence. I would rather say „Here, we use Drosophila to investigate the formation and remodeling of HTCA throughout spermiogenesis by visualizing key subunits of this complex".

Line 117 in Results section: „Thus the lengthening of the Centriole Cap IS DUE TO the centriole moving into the nuclear space rather than remaining fixed at a point and growing into the nucleus". I have problem with „is due to". The evidence (measuring the centrioles and proving they do not grow longer) is not so direct and strong to definitely rule out all other possibilities. I would soften this statement saying „... lengthening of the Centriole cap is most probably due to ..."

First revision

<u>Author response to reviewers' comments</u>

Response to Reviewers

We thank all of the reviewers for their insightful comments and enthusiastic responses to our work. The reviewers agreed that our work fills a significant gap in understanding the mechanism of sperm head-tail connection and centriole remodeling and provides a groundwork for future studies in male fertility. The reviewers asked for clarification on certain points and provided suggestions to modify the text so that our conclusions more accurately represent the data. In response, we have added a new representative image to Figure 2D and have made numerous revisions to the text. We describe the changes we've made to the manuscript in the point-by-point response below.

Reviewer 1

> *Advance Summary and Potential Significance to Field:*
>
> *Unlike somatic cells, sperm cells evolve atypical centrioles rapidly, and understanding the role of this atypical centriole is both timely and a priority if we are to better understand how sperm function. This paper provides an important and significant understanding of the role of the PCL, an atypical centriole found in insects in sperm*

© 2024. Published by The Company of Biologists under the terms of the Creative Commons Attribution License (https://creativecommons.org/licenses/by/4.0/).

head-neck architecture, and is, therefore, excellently suitable for publication after the minor corrections described below.

The paper shows that the fly sperm nucleus base is remodeled into a 'Centriole Cap' and 'Nuclear Shelf,' structures that resemble the basal plate in mammals. It also shows that the PCM (aka insects' centriole adjunct) remodeling is a parallel event, possibly allowing the giant centriole to penetrate the nucleus base. The paper also shows that the PCL is associated with the nuclear shelf moving along the GC as the GC invaginate the nucleus. Affecting the PCL by mutating POC1B or PLk4 RNAi resulted in the loss of maintaining GC invagination; however, the sperm neck maintained attached to the head.

An important emerging picture gained by this paper is that centriole remodeling is coupled to sperm head-neck coupling, paving the way to study if this link is conserved in other animal gropes, such as mammals. Interestingly, the PCL defect and POC1 did not break the connection of the head to the neck but affected the presence of neck/head attachment architecture.

We thank the reviewer for their enthusiastic and positive response to our work, and for highlighting how our study may impact future work in other model systems. We would like to point out, however, that loss of the PCL does lead to detachment of centriole from the nucleus at a low frequency (2% , see figure 5B).

Comments for the Author:

The title may be better changed from "The Proximal Centriole-Like Structure Anchors the Centriole to the Sperm Nucleus" to "The Proximal Centriole-Like Structure Maintains the Architecture of the Sperm Centriole—Nucleus Anchoring," as it is not clear to me that PCL's direct role in anchoring the Centriole to the Sperm Nucleus is established.

In the manuscript we document several classes of the centriole attachment defect seen the POC1 mutant and we hypothesize that they represent stages along the way to full detachment, which we see 2% of the time. While we do believe that the title accurately describes the data, we do agree that it does not describe the most prominent phenotype. We have revised the title to "The Proximal Centriole-Like Structure Maintains Nucleus-Centriole Architecture in Sperm."

The term HTCA is commonly used in mammal sperm, but the term sperm neck is more commonly used in fly research. Please add that HTCA is also known as sperm neck.

We thank the reviewer for pointing this out. We have included on line 53-54 that the HTCA is also referred to as the sperm neck.

To help a more general audience understand the paper, it will help to add that Spag4 is also known as SUN5 at the first time the protein is presented in the text. Also, indicate the mammalian orthologs of Drosophila proteins, such as Yuri Gagarin, and otherwise indicate if they are insect specific.

We agree that this additional context will be helpful for a general audience. We have now included all the known mammalian orthologs for the *Drosophila* proteins.

Please indicate the model system used to make a particular finding. For example - are these mice or fly: "Previous work discovered that initial attachment between the nucleus and centriole in Round spermatids is dependent on dynein at the nuclear envelope (Anderson 85 et al., 2009; Li et al., 2004; Sitaram et al., 2012)." Add that information throughout the paper.

We have updated the text to indicate the model system used for various findings referenced

© 2024. Published by The Company of Biologists under the terms of the Creative Commons Attribution License (https://creativecommons.org/licenses/by/4.0/).

throughout the paper.

The ”Centriole Cap” and “Nuclear Shelf” are reminiscent of the basal plate, and I wonder why the author does not prefer the term collectively as “basal plate-like.”

We thank the reviewer for pointing out that these structures resemble the basal plate, and we agree. We did not explicitly state this in the manuscript as we believed there are sufficient structural difference (based on EM) that would preclude the analogy. Namely, the basal plate is an electron dense thickening at the nuclear envelope, while no such thickening is evident in *Drosophila* (Tokuyasu, 1974). However, we agree that the Centriole Cap and Nuclear Shelf may functionally serve a similar purpose by serving as a platform for key HTCA proteins to link the sperm head and tail. Therefore, we have added to the discussion on lines 272-279 how the Centriole Cap and Nuclear Shelf may function as a “basal plate-like” structure.

You may want to address a recent paper in the current paper: Yunfei Zhang, Gang Liu, Lihua Huang, Xiyi He, Yuyan Su, Xinmin Nie, Zenghui Mao, Xiaowei Xing, SUN5 interacts with nuclear membrane LaminB1 and cytoskeletal GTPase Septin12 mediating the sperm head-and-tail junction, Molecular Human Reproduction, 2024;, gaae022, https://doi.org/10.1093/molehr/gaae022.

We thank the reviewer for pointing out this recent article. We have added discussion of this paper on lines 331- 336.

*****

Reviewer 2

Advance Summary and Potential Significance to Field:

The basal body-spermatid nucleus interaction is a vital aspect of animal spermiogenesis., since the correct positioning of the axoneme is directly correlated to its function during fertilization. Anormal basal body-spermatid nucleus interaction has been indeed correlated to infertility problems in humans.
However, how the basal body interacts with the nucleus during spermiogenesis progress has not been until now satisfying clarified.

Comments for the Author:

The main aim of the manuscript entitled “The proximal centriole-like structure anchors the centriole to the sperm nucleus“ is to investigate the dynamics of the basal body-nucleus interactions during the process of spermiogenesis in Drosophila melanogaster. The AA addressed this aspect by examining the localization on the centriole adjunct of specific proteins, i.e. Spag4, Yuri, Poc1, to clarify the role of these actors in the dynamics of the centriole adjunct and basal body anchoring and stabilization to the basal side of the sperm nucleus. In my opinion the present manuscript is interesting and deserves publication, since the problem addressed is a general aspect of the spermiogenesis that has so far been poorly understood. I have only a few marginal questions that could be easily addressed by the AA.

We thank the reviewer for their enthusiasm and positive comments and for highlighting that this area of work is understudied.

Minor comments

l.35-36. “Fertilization requires a properly formed sperm, which is comprised of a ‘head’ containing the genetic material and a ‘tail’ that drives sperm motility”. This is an general

© 2024. Published by The Company of Biologists under the terms of the Creative Commons Attribution License (https://creativecommons.org/licenses/by/4.0/).

condition, but there are several examples (insects, nematodes, etc.) in which the sperm are aflagellate and lack the axoneme.

We thank the reviewer for pointing this out and have added this caveat to lines 42-43.

It would be useful for readers if the AA describes the dynamics of the PCL. In l.163 the AA claim "This structure is known as the proximal centriole-like (PCL) structure, an atypical procentriole found in spermatids that serves as a template for daughter centriole formation during the first zygotic divisions. I think that the AA refer to the publish data, but it is unclear for me why the PCL is not found as distinct structure in mature sperm. The PCL is visible as incomplete procentriole during spermatid elongation of several insect species, but it is no longer visible as a structured organelle in mature sperm.

We thank the reviewer for pointing out this discrepancy. The reviewer is correct that the PCL is remodeled during spermiogenesis such that immuno-staining (or GFP tagging) of known PCL components do not identify a structure. However, the PCL is actually present and visible by EM in the mature spermatozoa of *Drosophila melanogaster*. This is shown in by Khire et al, 2016. We have modified the text on lines 178-180 to highlight this nuanced, but important point.

L. 119. "Thus the lengthening of the Centriole Cap is due to the centriole moving into the nuclear space rather than remaining fixed at a point and growing into the nucleus". Are there published data explaining the centriole movement?

To our knowledge, there is no published data on the mechanism by which the centriole moves into the nuclear space, nor evidence of daughter procentrioles moving along the long axis of the mother more generally. We have updated the text on lines 128-131 accordingly. We have also added additional discussion of the relationship between centriole invagination and PCL movement on lines 241-244, 282-284, and 291-293.

I agree with the AA that Spag4 have an internal localization with respect to Yuri that is a cytoplasmic proteins, but from the pictures it seems that the proteins colocalize on the nuclear envelope. It is very interesting! Please explain.

Due to limitations with our fluorophores and microscope resolution, and given that the nuclear envelope is only ~35nm in thickness, we are unable to resolve the precise localizations of Spag4 and Yuri to the inner nuclear envelope and cytosol, respectively. Thus, at this time, they do appear colocalized, but it is likely an artifact of low resolution. With future improved resolution, we hope to resolve this issue

Fig. 2D. The Asl staining is very weak and I can see two small Asl clusters close to the nucleus. Please explain.

Asl staining becomes dimmer in later stages of spermiogenesis as the centrioles are remodeled, hence its dimmer appearance in Fig 2D compared with the rest of Fig 2. The additional clusters of Asl near the nucleus in that image are Asl staining that corresponds to another spermatid in a different Z-plane. To avoid confusion, we have selected a new image for Fig 2D.

l. 206-208. "We therefore conclude that the PCL is not required for centriole invagination and lateralization, but is required for maintaining invagination of the centriole in late stages of spermiogenesis." It is unclear the relationship between PCL movement and basal body stabilization to the nucleus. Please, could the AA better explain?

We apologize to the reviewer for any confusion. Based on the fact that the PCL remains below the Nuclear Shelf as the basal body invaginates into the nucleus, we hypothesize that the PCL serves as an additional point of contact between the basal body and the HTCA to stabilize basal body

© 2024. Published by The Company of Biologists under the terms of the Creative Commons Attribution License (https://creativecommons.org/licenses/by/4.0/).

during invagination. It is possible that the PCL actively moves down the basal body, dragging the nuclear envelope with it to surround the basal body during invagination. However, invagination still occurs in *poc1* mutants (with no PCL). Thus, it is more likely that the PCL is simply passively anchored at the base of the Nuclear Shelf as the basal body actively moves into the nuclear space. We have added this additional context and discussion on lines 241-244, 282-284, and 291-293.

> *l. 286-289. "However, the CA remodelling could involve a phase transition centered around the PCL as it moves from its initial position at the proximal end of the centriole to its final position in the middle of the centriole, just below the Nuclear Shelf". I understand the hypothesis of the AA, but how the AA can exclude a passive movement of the PCL following CA remodelling? Are there data on PCL dynamics in mutants for proteins that affect the CA?*

The reviewer is correct that we cannot exclude the possibility that PCL dynamics and CA remodeling are not directly related to one another. Unfortunately we cannot test this idea directly as disrupting the CA using currently known components have severe upstream consequences on other aspects of spermatogenesis, including meiosis, centriole duplication, and microtubule dynamics. We have added this caveat on lines 316-317.

> *Fig 1S B,C The lateral position of the basal body at the needle stage is not so evident in immunofluorescence pictures.*

We agree that the lateral position of the centriole is not as obvious at the needle stage. Unfortunately, our levels of the basal body marker Ana1 is significantly reduced at this stage, making it difficult to image lateralization of the basal body using immunofluorescence. However, lateralization at these stages is evident by EM, as documented by Khire et al., 2016.

*****

Reviewer 3

> *Advance Summary and Potential Significance to Field:*
>
> *In this new and original research Buglak and colleagues investigated the mechanism by which the Head- Tail Coupling Apparatus (HTCA) secures the axoneme to the nucleus in elongating Drosophila spermatids. Using two prominent HTCA components, Spag4 and Yuri as markers they looked at the formation and stabilization of HTCA during spermatogenesis and they did a nice morphological characterization by super-resolution microscopy. Seeking for mechanism involved in remodeling of HTCA, they demonstrated a correlation between condensation of the centriole adjunct (CA, marked with Asterless antibody) and the invagination of the centriole coupled with HTCA remodeling. However, as authors correctly admit, it is difficult to reveal the direct mechanism with classical genetic tools as asterless mutants affect centriole duplication and microtubule nucleation as well. Moreover, using Sas6 and Ana1 as markers, they investigated the role of procentriole-like structure (PCL) in the context of HTCA formation. The mechanistic glimpse comes from poc1B mutant lacking proper PCL, and the conclusion is that PCL is required in stabilization of the final head-tail connection.*
>
> *As a conclusion, we see here a work which provides new and original scientific knowledge, lays a good foundation for further mechanistic HTCA research, and it has a potential to be interesting for a wider scientific audience, including broad research areas working on male fertility. It already gives an explanation why abnormal level of POC1B could cause sperms with abnormal morphology and unexplained infertility in humans.*

We thank the reviewer for their insightful and positive comments and for highlighting that our work provides a foundation for future HTCA research.

© 2024. Published by The Company of Biologists under the terms of the Creative Commons Attribution License (https://creativecommons.org/licenses/by/4.0/).

Comments for the Author:

In the abstract at line 24 there is statement „high-resolution map of proteins and structures at the HTCA". This line promises to much, generates and expectation which is not satisfied by the article itself. Based on this the reader somehow expect proteomics done in an attempt to find all HTCA components strengthen by protein-protein interaction maps and they localization in the mutants of each other. Given the fact that only two HTCA proteins, Spag4 and Yuri were investigated, I would soften this sentence. I would rather say „Here, we use Drosophila to investigate the formation and remodeling of HTCA throughout spermiogenesis by visualizing key subunits of this complex".

We agree with the reviewer's point and have amended the statement in the abstract on lines 24-25 as suggested.

Line 117 in Results section: „Thus the lengthening of the Centriole Cap IS DUE TO the centriole moving into the nuclear space rather than remaining fixed at a point and growing into the nucleus". I have problem with „is due to". The evidence (measuring the centrioles and proving they do not grow longer) is not so direct and strong to definitely rule out all other possibilities. I would soften this statement saying „... lengthening of the Centriole cap is most probably due to ..."

We agree that the evidence provided is not sufficient to state that cap lengthening is due to the centriole moving into the nuclear space. We have amended this statement on line 127 as suggested.

Second decision letter

MS ID#: JOCES/2024/262311

MS TITLE: The Proximal Centriole-Like Structure Maintains Nucleus-Centriole Architecture in Sperm

AUTHORS: Danielle B Buglak, Kathleen H Holmes, Brian J Galletta, and Nasser M Rusan
ARTICLE TYPE: Research Article

I am happy to tell you that your manuscript has been accepted for publication in Journal of Cell Science, pending standard publication integrity checks.

© 2024. Published by The Company of Biologists under the terms of the Creative Commons Attribution License (https://creativecommons.org/licenses/by/4.0/).
